# Cytotoxicity Test of One-Step Self-Etching Bonding Agents by Standardized Dentin Barrier Test Using Polyurethane Discs

**DOI:** 10.3390/ma7010085

**Published:** 2013-12-23

**Authors:** Mi-Joo Kim, Kyoung-Nam Kim, Kwang-Mahn Kim

**Affiliations:** Department and Research Institute of Dental Biomaterials and Bioengineering, Yonsei University College of Dentistry, 50 Yonsei-ro, Seodaemun-gu, Seoul 120-752, Korea; E-Mails: vagusmj@yuhs.ac (M.-J.K.); kimkn@yuhs.ac (K.-N.K.)

**Keywords:** cytotoxicity test, dentin barrier test, dentin substitute, one-step self-etching bonding agent, perfusion cell culture

## Abstract

This study was performed to standardize a dentin barrier test with the substitute and evaluate the cytotoxicity of one-step self-etching bonding agents. Each of the natural bovine dentin and polyurethane discs were 500-μm thick and were tested using a perfusion device. Following the treatment with 0.05% phenol on the natural bovine disc or three kinds of polyurethane discs—30, 40, and 50 pcf (pounds per cubic foot)—cell viability of L-929 was measured by the 3-(4,5-dimethylthiazol-2-yl)-2,5-diphenyltetrazolium bromide (MTT) assay and expressed as percentages of non-treated group, respectively. A substitute showing permeability similar to that of bovine dentin was determined based on this result. Cytotoxicity test of bonding agents was performed by the selected substitute, the results of which were expressed as percentages of the control. In addition, SEM images were taken after the tests. The cell viability by 40-pcf polyurethane disc was not statistically different from that by bovine dentin disc (*P* > 0.05). Futurabond DC resulted in the highest cell viability and Bond force the lowest by the 40-pcf polyurethane disc (*P* < 0.05). The adhesives on the 40-pcf polyurethane disc changed cellular morphology with different degrees on the SEM images. This standardized test might be useful for assessing the cytotoxicity of dental materials applied to dentin before clinical applications.

## 1. Introduction

Biocompatibility in dentistry is defined as the capacity of a material to fulfill its function for a specific application with an appropriate response in the recipient of dental materials (from ISO 1942 [1]). This property has been extensively studied, because it is essential for ensuring both the safe treatment of patients and the practitioner’s health. Since most biomaterials used in dentistry have a direct or indirect effect on adjacent cells or tissue, *in vitro* and *in vivo* toxicity tests are indispensible prior to clinical applications of dental materials to humans.

However, there is an increasing need for alternative test models that are more reproducible and efficient than animal experiments and clinical studies because these toxicity tests for dental materials are time-consuming, expensive, and subject to extensive public discussions. As an alternative, *in vitro* cell culture methods have several important technical advantages compared with *in vivo* tests; they are better standardized, more reproducible, and faster and easier to perform at relatively low costs [2–4]. Therefore, many researchers are working to find and develop substitution methods to replace animal experiments in dentistry: to illustrate, dentin barrier test, murine local lymph node assay (LLNA), bovine corneal opacity and permeability (BCOP) assay, skin irritation alternative test method using human cells, and other biomimetic cytotoxicity tests are now used [5–9]. In particular, a dentin barrier test is a suitable evaluation method simulating the *in vivo* cytotoxic effects of dental filling materials, luting cements, or therapeutic regenerative materials in the restoration, cementing or other application procedures after tooth preparation. This can mimic clinical situations better than direct cell–material contact, and it has the potential to replace *in vivo* experimentation [10–12].

In a dentin barrier test, natural bovine teeth are generally used instead of human teeth; they are easier to obtain and have morphological characteristics similar to human teeth [13,14]. However, since bovine teeth are natural, individual-dependent experimental variations exist whenever this test is performed [15]. Because the permeability of dentin plays a key role in influencing the diffusion of the released toxic materials through dentin to the pulp, standardization of this point can be a critical factor to get a constant result from the dentin barrier test.

In the present study, we substituted natural dentin barriers with artificial, more standardized ones to attain more controllable results from *in vitro* cytotoxicity tests. Thus, polyurethane foam was used as the dentin substitute. This material is originally used for testing orthopedic implants, instruments, and other educational models. Because polyurethane foam has a porous structure with different degrees, is easily controlled by various commercial processes, and can be made into a thin disc-shaped product, this has the potential to mimic the natural dentin structure in a dentin barrier test. Therefore, the aim was to find a biocompatible dentin substitute with permeability similar to that of natural tooth so as to standardize these conditions in a dentin barrier test and perform a cytotoxicity test of six kinds of one-step self-etching bonding agents by the substitute.

## 2. Results and Discussion

### 2.1. Cell Viability Test

#### 2.1.1. MTT Assay with Phenol

**Figure 1.**
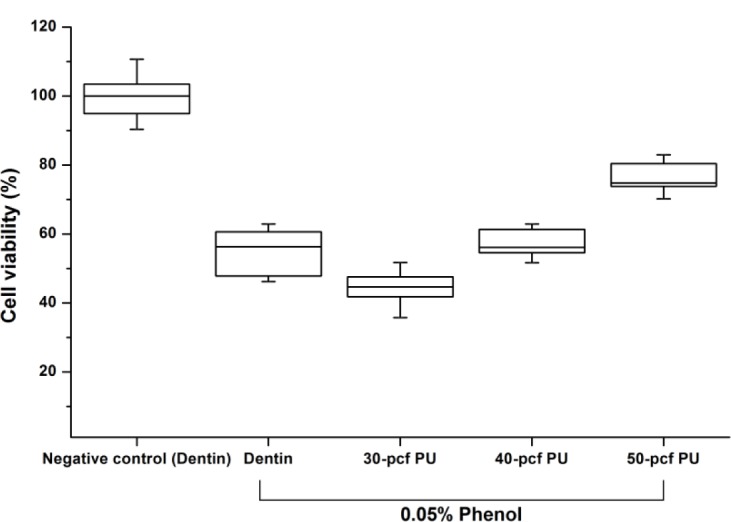
Cell viabilities of 0.05% phenol dilution by bovine dentin or three types of polyurethane discs. This figure shows the cytotoxicity test of 0.05% phenol by bovine dentin or polyurethane discs. Each box in the boxplot graph means the maximum, 75%; median, 25%; and minimum values. Cell viability of negative control (non-treated group)with dentin was set as 100% cell viability. Dentin disc treated by 20 μL of 0.05% phenol made approximately 50% cell viability. The cell viability with polyurethane discs increased according to the increases of disc density (*P* < 0.05). The 40-pcf polyurethane disc was not statistically different from natural dentin disc (*P* > 0.05). Groups with different letters above the data bar are statistically significant (*P* < 0.05).

Figure 1 shows the cell viability of dentin or polyurethane disc with 0.05% phenol concentration. Cell viability expressed as percentages of the control group was increased by the increase in disc density (*P* < 0.05). The 40-pcf (pounds per cubic foot) polyurethane disc was not statistically different from the dentin disc (*P* > 0.05), and therefore, it was selected as a dentin substitute in the following experiment.

#### 2.1.2. MTT Assay with One-Step Self-Etching Bonding Agents

**Figure 2.**
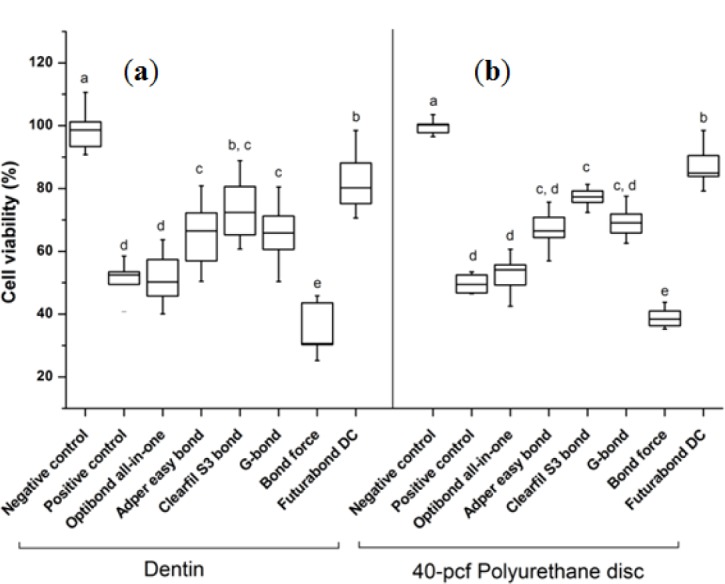
Cell viabilities of different self-etch adhesives using bovine dentin or 40-pcf polyurethane disc as a barrier. This figure shows the cytotoxicity test of different self-etch adhesives by (**a**) bovine dentin; or (**b**) 40-pcf polyurethane disc. Each box in the boxplot graph means the maximum, 75%; median, 25%; and minimum values. Among adhesives, Futurabond DC showed the highest cell viability, and Bond force did the lowest one (P < 0.05) regardless of the barrier type. The gap of standard deviations by 40-pcf polyurethane disc was less than that by natural bovine dentin in all groups. Groups with different letters above the data bar are statistically significant (*P* < 0.05).

The cytotoxicity results of six kinds of one-step self-etching bonding agents by bovine dentin or 40-pcf polyurethane discs are summarized in Figure 2. Bond force showed the lowest cell viability and Futurabond DC the highest among the six agents regardless of the barrier type (*P* < 0.05). The gap of standard deviations by 40-pcf polyurethane disc was less than that by bovine dentin in all groups.

### 2.2. SEM Images after the Application of Dentin Bonding Agents

**Figure 3.**
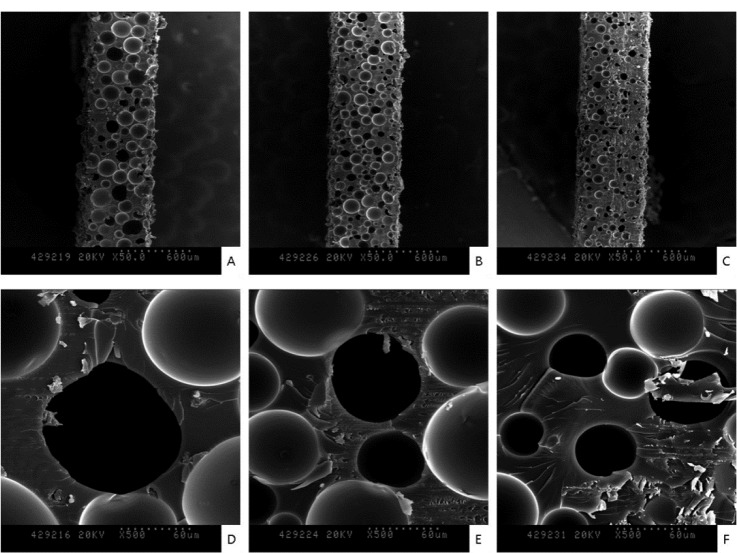
SEM images of 500-μm thick, 30, 40, and 50-pcf polyurethane discs. (**A***,***D**) The 30-pcf polyurethane disc (50× and 500×). (**B**,**E**) The 40-pcf polyurethane disc (50× and 500×). (**C**,**F**) The 50-pcf polyurethane disc (50× and 500×).

**Figure 4.**
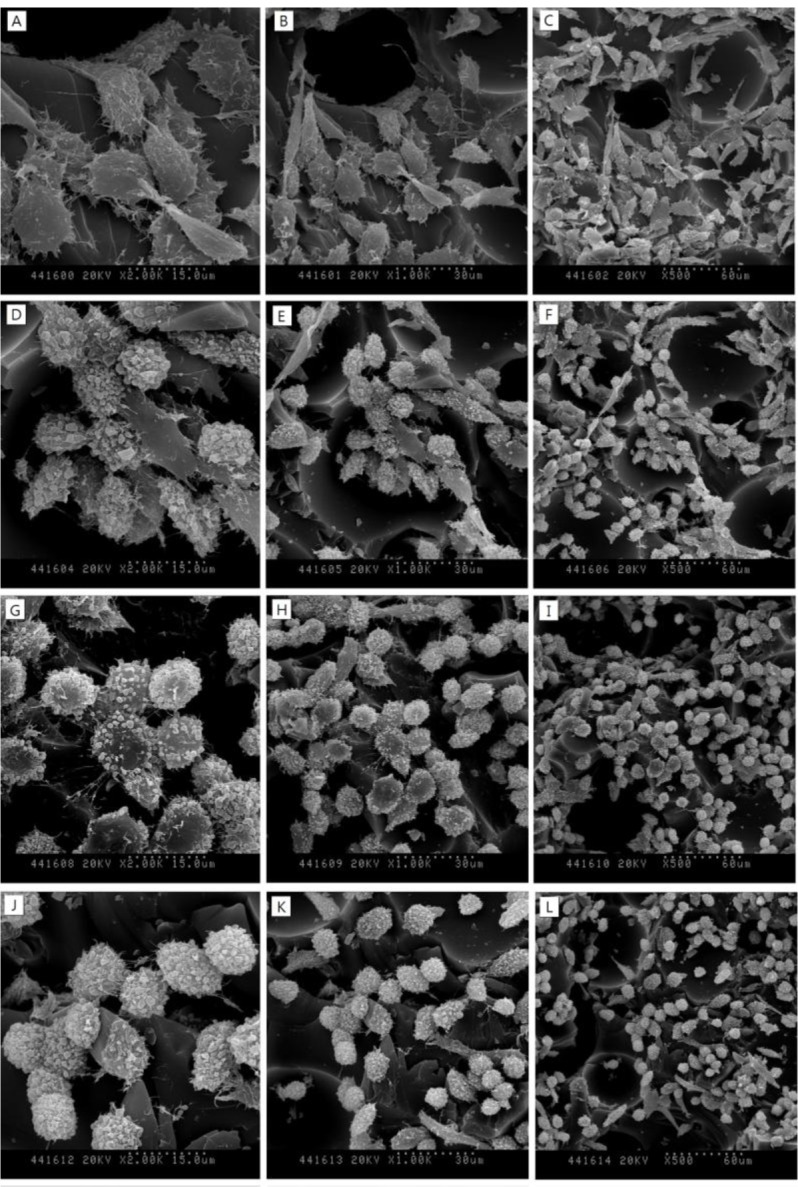

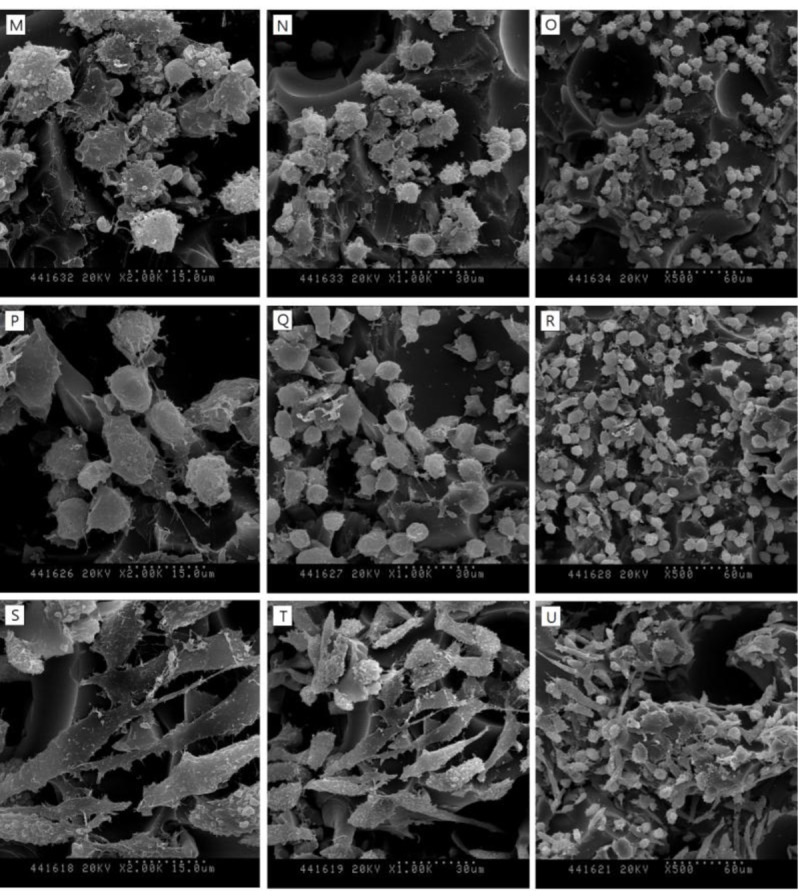
SEM images of cellular changes on 40-pcf polyurethane discs with different phenol concentration. (**A**–**C**) Negative control (2000×, 1000× and 500×); (**D**–**F**) Optibond all-in-one (2000×, 1000× and 500×); (**G**–**I**) Adper easy bond (2000×, 1000× and 500×); (**J**–**L**) Clearfil S^3^ bond (2000×, 1000× and 500×); (**M**–**O**) G-bond (2000×, 1000× and 500×); (**P**–**R**) Bond force (2000×, 1000× and 500×); (**S**–**U**) Futurabond DC (2000×, 1000× and 500×) on the 40-pcf polyurethane disc. The self-etch adhesives changed cell morphology with globular shape or torn surface, whereas L-929 cells freely extended their branches along the pores in the negative control (**A**–**C**). Especially, each 2000× magnification showed cellular destruction and cracks well.

SEM images in Figure 3 show the surfaces of 30, 40 and 50-pcf polyurethane foam (A, D; 30-pcf, B, E; 40-pcf, C, F; 50-pcf, sectioned polyurethane foam). Figure 4 describes the morphological features of cells on the 40-pcf polyurethane disc with each dentin bonding agent, respectively (D–U), which were compared with the control group (A–C). L-929 cells could be cultured easily, forming direct contacts on the polyurethane discs in the control group without toxic materials (Figure 4A–C, 2000×, 1000× and 500×, respectively). Bond force induced severe cellular destruction and subsequently, surface cleavage, smooth surfaces and globular morphology in Figure 4P–R. The differences among Optibond all-in-one (Figure 4D–F), Adper easy bond (Figure 4G–I), Clearfil S^3^ bond (Figure 4J–L) and G-bond (Figure 4M–O) could not be distinguished from one another in Figure 4, although there were statistical differences in MTT assay. Otherwise, the control group did not show negative effects on the cells attached to the polyurethane disc and had normal cellular adhesion (Figure 4A–C). Comparatively, Futurabond DC had the highest optical density in Figure 2, which was in line with the SEM images in Figure 4S–U. Most cells had comparatively long body for good adhesion, while a small proportion of cells with spherical shape existed on the 40-pcf polyurethane disc when combined with Futurabond DC.

The dentin disc used in a dentin barrier test is an effective barrier, preventing cell damage from a great variety of materials and chemicals. The diffusion of chemical products or other substances from dentin to the pulp is dependent upon several important variables, including (a) the thickness of the remaining dentin—the thicker the dentin, the lower the concentration of microbial products [16]; (b) the surface area of the exposed, permeable dentin—a thin, crescent-shaped area of exposed cervical dentin permits much less permeation than a leaking full-crown preparation [17,18]; (c) the presence or absence of a smear layer [12]; (d) the potency of the microbial products [19]; and (e) the rate of pulpal blood flow [20]. Among these variables, thickness of remaining dentin and smear layer can be controlled by a production process, the surface area of the exposed dentin by standardized barriers, and pulp blood flow by an artificial perfusion system. Based on these facts, controlling porous barrier surfaces and mimicking the pulp flow were the key factors examined in this study. Polyurethane foam is a commercial product with uniform and consistent physical properties; it is often used as a replacement for human cadaver bone in biomechanical tests of orthopedic implants [21–24]. In addition, polyurethane foam can also be utilized as a porous and stable substitute with retention of its features and penetration of toxic agents. Therefore, we chose this material as the dentin substitute in a dentin barrier test.

The effects of toxic agents are selective and dependent on the chemical nature of the dentin-contacting material [6,18,25]; therefore, the variables like viscosity, acidity, and other factors that depend on the chemicals should be removed for the purpose of finding the dentin substitute with characteristics similar to natural dentin discs. We used dilutions of phenol and water, as recommended in ISO 10993-12 [26], and initially tested these as standard positive control. Adaptations of various materials as dentin bonding agents would be a process to initiate after finding the dentin substitute has similar permeability to natural dentin.

One-step self-etching, seventh-generation bonding agents were introduced in the mid 2000s, and both the light-cured and dual-cured systems are available. The light-cured products typically require one step, whereas the dual-cured products typically require two steps—chemical and light-curing. Among the six kinds of bonding materials, Futurabond DC showed the highest cell viability in MTT assay and comparatively biocompatible features on the SEM images. Futurabond DC is a dual-curing type in that two kinds of liquid are mixed right before application, indicating that this material can be polymerized in advance of light-curing by chemical reaction and that additional chemical curing is available on the surface of adherent after light-curing, leading to more complete polymerization. Therefore, in clinics, it is possible that dual-cured type of adhesives would be less toxic to the cells and tissue despite the comparatively short shelf time and working time after mixing, which are the shortcomings of these materials. For more definite conclusions, diverse kinds of self-etch dual-cure adhesives should be compared with one another in further studies.

In this study, we used foams of three different densities—30, 40, and 50-pcf as shown in Figure 3 and performed cytotoxicity test of bonding agents. Cellular reactions to the 40-pcf polyurethane foams are illustrated in Figure 4; post-attachment cell morphology (A–C, control) shows that L-929 cells freely extended their branches along the pores, indicating the stability of polyurethane discs as the dentin substitute. However, toxic materials changed cell morphology into globular shape or torn flat surface, especially in Bond force group (P–R). Comparatively, Futurabond DC was less toxic than the others, and this was in accord with the cytotoxicity test results in Figure 2.

Polyurethane foam has an advantage in making a large quantity of standardized commercial discs and testing various toxic materials easily. However, the compositions and microstructures are different between polyurethane discs and bovine or human teeth. Major components of dentin include an inorganic reinforcing phase of apatite (about 50% by vol.) and an organic matrix mainly composed of type I collagen (approximately ~30% by vol.) [27]. If test materials pass through natural dentinal tubules (2.4–4.8 μm in diameter [28]), toxicity induced by applied ones can be neutralized by reactions with these organic or inorganic components. Otherwise, this phenomenon cannot be simulated by the present model because of the foaming microstructure of the polyurethane disc.

Despite such limitations, this investigation represents a significant step toward reducing variations of *in vitro* tests by controlling permeability, suggesting that biocompatible polyurethane foam could be a standardized barrier substitute—40-pcf polyurethane disc—for use in the dentin barrier test. In particular, this standardized system would be required to evaluate the biocompatibility of dental commercial products applied to dentin indirectly in clinics. Establishment of the dentin substitute can surely reduce the needs for expensive and time-consuming *in vivo* experiments and animal tests, leading to the development of more standardized evaluation methods and new innovative biocompatible products.

## 3. Experimental Section

### 3.1. Dentin and Substitute Disc Preparation

Dentin discs of 500-μm thickness and 13-mm diameter were cut from bovine incisors and polished using sandpaper of #100-#1200 grit. The disc surfaces were etched for 30 s with 50% citric acid, as previously described [29]. For the dentin substitute, we used 500-μm thick discs of polyurethane foams of three different densities (Sawbones, Washington, DC, USA): 30-pcf (0.48 g/cc; product number 1522-04), 40-pcf (0.64 g/cc; 1522-05), and 50-pcf (0.80 g/cc; 1522-27). Foam densities designate “graded” foams according to ASTM standard specification F-1839-08. Each dentin or polyurethane disc was inserted into a Minusheet (Minucells and Minutissue, Bad Abbach, Germany) made of two donut-shaped rings so that the disc could be placed between them.

### 3.2. Cell Culture on Dentin or Substitute

The dentin or substitute was coated with 0.03 mg/mL fibronectin (Sigma-Aldrich, St. Louis, MO, USA) in sterile water and dried for 2 h in a dry cabinet before being seeded with 40 μL (2.5 × 10^5^ cells/mL) L-929 mouse fibroblasts. The medium used for cell culture was RPMI 1640 supplemented with 10% fetal bovine serum (FBS), 150 IU/mL penicillin, 150 μg/mL streptomycin, 0.125 μg/mL amphotericin B, and 0.1 mg/mL geneticin (Gibco, Grand Island, NE, USA). After cell adhesion occurred, 2 mL growth medium was added to each well and the cells were cultured for 72 h at 37 °C in a humidified atmosphere containing 5% CO_2_.

### 3.3. Cytotoxicity Testing Using *In Vitro* Perfusion Chamber System

After 72 h of cultivation with cells, the dentin or polyurethane discs were transferred to a perfusion culture system (Minucells and Minutissue, Bad Abbach, Germany) connected to a perfusion pump (Ismatek, Woking, UK) that could adjust the flow of medium. The cells attached to the dentin or substitute surface were located on the lower side (“pulpal” side) in the chamber. All culture chambers were set on a 37 °C warm plate to maintain a constant temperature. The initial medium flow rate was 0.3 mL/h for 24 h. After 24 h, 20 μL of toxic materials (one-step self-adhesives or 0.5% phenol) quantified by the micropipette were gently applied to the upper surface of the dentin or polyurethane disc, and the flow rate was increased to 2 mL/h to simulate the *in vivo* environment for 24 h. All chambers were perfused with a culture medium containing 5.96 g/L HEPES buffer (Sigma-Aldrich, St. Louis, MO, USA). Negative controls without test materials were prepared and tested in the same way.

First, cytotoxicity test of 0.05% phenol (determined by the preliminary study), one of the representative positive controls recommended in ISO 10993-12 [26], was performed using the 500 μm bovine dentin disc or three kinds of polyurethane discs. This was the procedure to determine the dentin substitute showing approximately 50% cell viability by phenol and similar permeability to natural bovine dentin. Second, the cell viability percentage of each dentin bonding agent by the selected substitute was compared with one another. Bonding agents used for the experiment include Optibond all-in-one (Kerr, Tokyo, Japan), Adper easy bond (3M ESPE, St. Paul, MN, USA), Clearfil S^3^ bond (Kuraray medical Inc., Okayama, Japan), G-bond (GC, Tokyo, Japan), Bond force (Tokuyama, Tokyo, Japan) and Futurabond DC (Voco, Cuxhaven, Germany). After applying the bonding agents, light was irradiated using a light-curing unit—Elipar Freelight 2 (3M ESPE, St. Paul, MN, USA, 1200 mW/cm^2^) —according to the manufacturer’s instructions to mimic clinical situations. Table 1 shows commercial products and their components used in this study. The cytotoxicity test of diverse toxic materials with either dentin or selected polyurethane disc was performed one at a time under the same conditions, and this procedure was repeated 10 times independently.

### 3.4. Measuring Cell Viability Using the MTT Assay

MTT (3-(4,5-dimethylthiazol-2-yl)-2,5-diphenyltetrazolium bromide) assay was employed to evaluate the cell viability of various toxic materials when using dentin or polyurethane discs. The absorbance at 540 nm was determined spectrophotometrically. The mean optical densities of 0.05% phenol or adhesives by the dentin or polyurethane disc were expressed as percentages compared with the values by the negative control (non-treated group), respectively.

### 3.5. Scanning Electron Microscopy (SEM) Analysis

SEM images were taken of the 30, 40, and 50-pcf polyurethane discs and the selected substitute surface after applying six kinds of one-step self-etching adhesives to visualize cell attachments on the dentin substitutes. Then, these were compared with the images from the control group. First, cells were fixed by using 2% paraformaldehyde and air-dried at room temperature for 1 h. Visualization of the cells on polyurethane discs was performed by using a Karl Zeiss EVO 40 model SEM instrument (Dresden, Germany). The gold layer coating (10-nm thickness) was performed by using a sputter coater (Model BAL-TEC SCD 005 Sputter Coater, Balzers, Liechtenstein) to impart electrical conductivity. The accelerating voltage was 5 kV for all experiments. SEM images were taken three times in each group. These were obtained from specific areas of interest at various magnifications.

### 3.6. Statistical Analysis

Statistical analysis was performed using One-way ANOVA (*P* > 0.05). Pair-wise multiple comparisons were carried out using the Tukey test when one-way analysis of variance detected a significant difference. *P* value < 0.05 was considered significant.

## 4. Conclusions

As a dentin substitute, the 40-pcf polyurethane disc with a standard toxic material had permeability similar to natural bovine dentin discs of the same thickness. MTT assay results and morphological analysis after the application of one-step self-etching bonding agents revealed cellular changes by toxic agents, and showed that polyurethane discs could be used as stable substitutes in a dentin barrier test. This method will be useful for evaluating the toxicity of dental materials applied to dentin.

## Figures and Tables

**Table 1.** Commercial self-etch adhesives and their compositions.

Product	Manufacturer	Lot Number	Ingredients	Application procedure
Optibond All-In-One	Kerr	3648431	methacrylate ester (33%–43%), ethyl alcohol (4%–9%), water, acetone (35%–45%), monomers, inert mineral fillers, ytterbium fluoride, photoinitiators, accelerators and stabilizers	apply adhesive for 20 s, air-dry, light-curing 10 s
Adper Easy Bond	3M ESPE	403636	methacrylated phosphoric esters, bis-GMA, camphorquinone, water, HEMA, polyalkenoic acid	apply adhesive for 20 s, air-dry, light-curing for10 s
Clearfil S^3^ Bond	Kuraray	051552	MDP, Bis-GMA, HEMA, camphorquinone, hydrophobic dimethacrylate, ethanol (<20%), water, silanated colloidal silica	apply adhesive for 20 s, air-dry, light-curing 10 s
G-bond	GC	1101221	4-MET, UDMA, silica, phosphoric acid ester monomer, acetone, water, photoinitiator	apply adhesive and wait for 5 to 10 s, air-dry, light-curing 10 s (LED light)
Bond force	Tokuyama	036E00	alcohol, phosphoric acid monomer, HEMA, Bis-GMA, TEGDMA, camphorquinone, purified water	apply adhesive for 20 s, air-dry, light-curing for 10 s
Futurabond DC	Voco	1024417	Bis-GMA, HEMA, BHT, ethanol, fluorides, camphorquinone, siliciumdioxide nanoparticles	mix a liquid A & B(1:1) for 2 s, apply adhesive for 20 s, air-dry, light curing for 10 s

Notes: Bis-GMA: bisphenol A diglycidyl methacrylate; HEMA: 2-hydroxyethyl methacrylate; BHT: Butylated hydroxy toluene; PEM-F: pentamethacryloyloxyethylcyclohexaphosphazene monofluoride; UDMA: urethane dimethacrylate; MDP: 10-Methacryloyloxydecyl dihydrogen phosphate.
